# Is the contralateral lesser trochanter a reliable reference for planning of total hip arthroplasty – a 3-dimensional analysis

**DOI:** 10.1186/s12891-021-04131-w

**Published:** 2021-03-11

**Authors:** Julian Hasler, Armando Hoch, Philipp Fürnstahl, Jakob Ackermann, Patrick O. Zingg, Lazaros Vlachopoulos

**Affiliations:** 1 Department of Orthopaedics, Balgrist University Hospital, University of Zurich, Forchstrasse 340, CH-8008 Zurich, Switzerland; 2 Research in Orthopaedics Computer Science, Balgrist University Hospital, University of Zurich, Zurich, Switzerland

**Keywords:** 3-Dimensional analysis, Lesser trochanter, Center of femoral head, Distance, Side‐to‐side difference, Total hip arthroplasty

## Abstract

**Background:**

Preoperative templating in total hip arthroplasty (THA) is mandatory to achieve appropriate offset and leg length equality. However, templating methods using the contralateral hip might be susceptible to errors resulting from side-differences in the femoral morphology. The distance of the lesser trochanter to the femoral head center (LTFHD) is a frequently used reference parameter for preoperative planning and intraoperative validation during THA. However, currently no three-dimensional (3D) analysis of side differences of the LTFHD exists.

**Methods:**

Using Computer tomography (CT)-based surface models from 100 paired femora (50 cadavers), side-to-side asymmetry of the LTFHD, femoral length, femoral head diameter (FHD) and femoral antetorsion were analyzed. Univariate linear regression models were established to evaluate potential associations between sides regarding LTFHD and FHD as well as a correlation of these parameters with each other.

**Results:**

Statistically significant side-differences were found for the LTFHD (*p* = 0.02) and FHD (*p* = 0.03) with a mean absolute side-difference of 1.6 ± 1.4mm (range 0.1–5.5mm) and 0.4mm ± 0.6mm (range 0–3mm), respectively. The ratio between the LTFHD and FHD was consistent with an average value of 1.16 ± 0.08 and reliable between sides with a correlation coefficient (r) of 0.72 (*p* < 0.01).

**Conclusions:**

The LTFHD is a reliable reference parameter for preoperative templating and intraoperative validation during THA with a high correlation between sides (*r* = 0.93, *p* < 0.01). However, 8 % of the investigated specimens revealed a LTFHD of more than 4mm, which should be anticipated during THA to avoid unsatisfiable results.

## Introduction

Restoration of the native biomechanical setting in total hip replacements is crucial for achieving complete functional recovery, stability and good clinical outcomes [1, 2]. To attain appropriate offset and limb-length equality, preoperative templating of total hip arthroplasty (THA) is mandatory [3–7]. The distance between the lesser trochanter and the center of the femoral head (LTFHD) is a frequently used reference for preoperative planning and intraoperative validation to restore normal anatomy of the proximal femur and the hip [8–10]. However, due to altered anatomy and therefore distorted radiological representation of the proximal femur in severely arthritic hips, femoral neck fractures or collapse of the femoral head arising from osteonecrosis of the hip, the preoperative radiographic assessment of limp length and offset of the affected side might be of limited value [11].Thus, the unaffected hip is often used to plan and evaluate accuracy of hip reconstruction [4, 12, 13]. Furthermore, some authors propose the femoral head diameter as a valuable tool to predict the LTFHD [14–16], which might be helpful if pathological conditions exist on both hips. However, these templating methods based on plane radiographs may be susceptible to errors resulting from projection errors [17, 18] and side-to-side differences of the 3-dimensional geometry of the proximal femur [19–21].

While it is common practice to utilize the contralateral side for THA planning, only limited data exists regarding side-to-side variability of the LTFHD [14]. The current study, therefore, sought to establish a 3D analysis of the side-to-side difference of the LTFHD among individuals with normal anatomy of the proximal femur on full body CT. Understanding of normal variations of this commonly used parameter might improve the accuracy of preoperative planning in THA.

## Materials and Methods

This study was approved by the institutional review board and the ethical committee (ID 2018–02242). CT full-body data of 50 cadaveric specimens provided by the Institute of Forensic Medicine, University of Zürich, Switzerland were included. There were 35 male and 15 female donors with an average age of 57 ± 20 years (median 58 years; range 18–86 years). The average weight was 71 ± 16 kg (median: 68 kg; range: 41 to 110 kg), the average height 171.5 ± 15.9 cm (median: 172 cm; range 137 to 190 cm) and the average BMI was 24.1 ± 5.2 kg/m^2^ (median: 22.7 kg/m^2^; range: 17.3 to 44.8 kg/m^2^). Exclusion criteria comprised any macroscopic pathology of the proximal femur visible on preoperative CT scan such as sequelae of childhood hip disorders including Legg-Calvé-Perthes disease or slipped capital femoral epiphysis, malunion or femoral head collapse arising from osteonecrosis of the hip, as well as advanced degenerative changes of the hip.

### Generation of 3D triangular surface models

CT full-body data of the included 50 cadaveric specimens were used to create 3D triangular surface models of 100 paired femora (50 right, 50 left). CT data were acquired using a Somatom Definition Flash CT scanner (Siemens Helathineers, Erlangen, Germany) with slice thickness of 1 mm. In order to create 3D triangular surface models, segmentation of the femora was conducted using the global thresholding and region growing functionality of a standard segmentation software (Mimics Medical, Materialise NV, Leuven, Belgium). For further analysis, the generated surface models were imported into the planning software CASPA (Computer Assisted Surgery Planning Application), which was developed at Balgrist University Hospital (Balgrist CARD [Computer Assisted Research and Development]).

### Definition of anatomical landmarks and parameters

Defining the following landmarks was warranted to quantify the femoral anatomy. First, we calculated an oriented bounding box of the whole femoral model per principal component analysis [22], thereby deriving a local coordinate system as described by Jud et al [23]. The length of the longest side of the oriented bounding box was defined as the femoral length.

For the quantification of the femoral antetorsion, we defined the perpendicular axis of the posterior condyles (PPC) and the true femoral neck axis (FNA) [24].

**Fig. 1:**
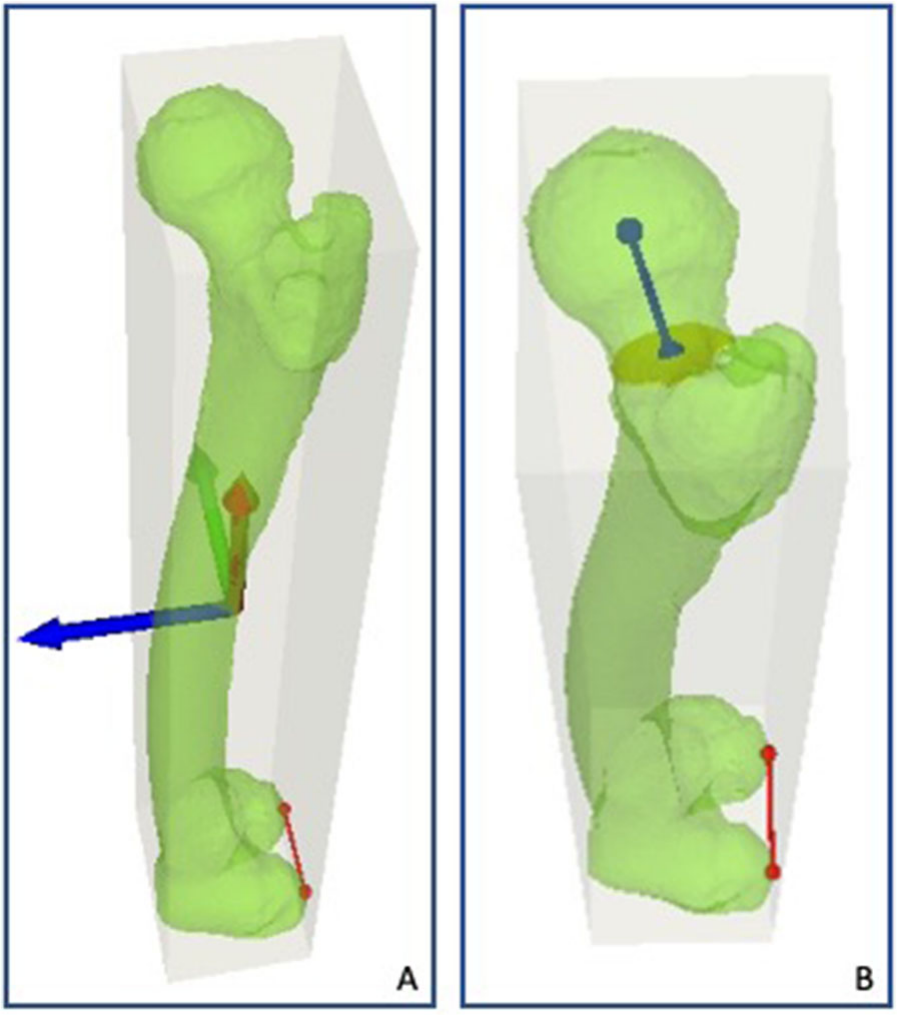
Measurement of femoral antetorsion. **a** Red line: Perpendicular axis of the posterior condyles (PPC). **b** Blue line: Femoral neck axis (FNA), defined as the axis between the femoral neck center (FNC) and the femoral head center (FHC). Femoral antetorsion was defined as the angle between the PPC and the FNA, both projected on the xy-plane of the coordinate system

For this purpose, the oriented bounding box was rotated around the z-axis of this coordinate system and adjusted in its anteroposterior expansion until the most posterior points on the medial and lateral femoral condyles were identified. The axis connecting these points yields the PPC (Fig. 1 A). For definition of the true femoral neck axis (FNA), the femoral head center (FHC) was defined as the center of a best-fit sphere calculated form all surface points of the femoral head. Thereafter, a cylinder was fitted into the femoral neck with the FHC as center of the cylinder. The axis of the cylinder is called hereafter preliminary neck axis (PNA). A plane - preliminary neck plane (PNP) - with the PNA as plane normal was defined with a distance of 1.25 times the femoral head radius from the FHC. The intersection of the femoral model and the PNP was used to calculate the femoral neck center (FNC), as the center of mass of these points. The FNA, was defined as the axis between the FNC and the FHC. Femoral antetorsion was then defined as the angle between the PPC and the FNA, both projected on the xy-plane of the coordinate system. (Fig. 1B)

**Fig. 2:**
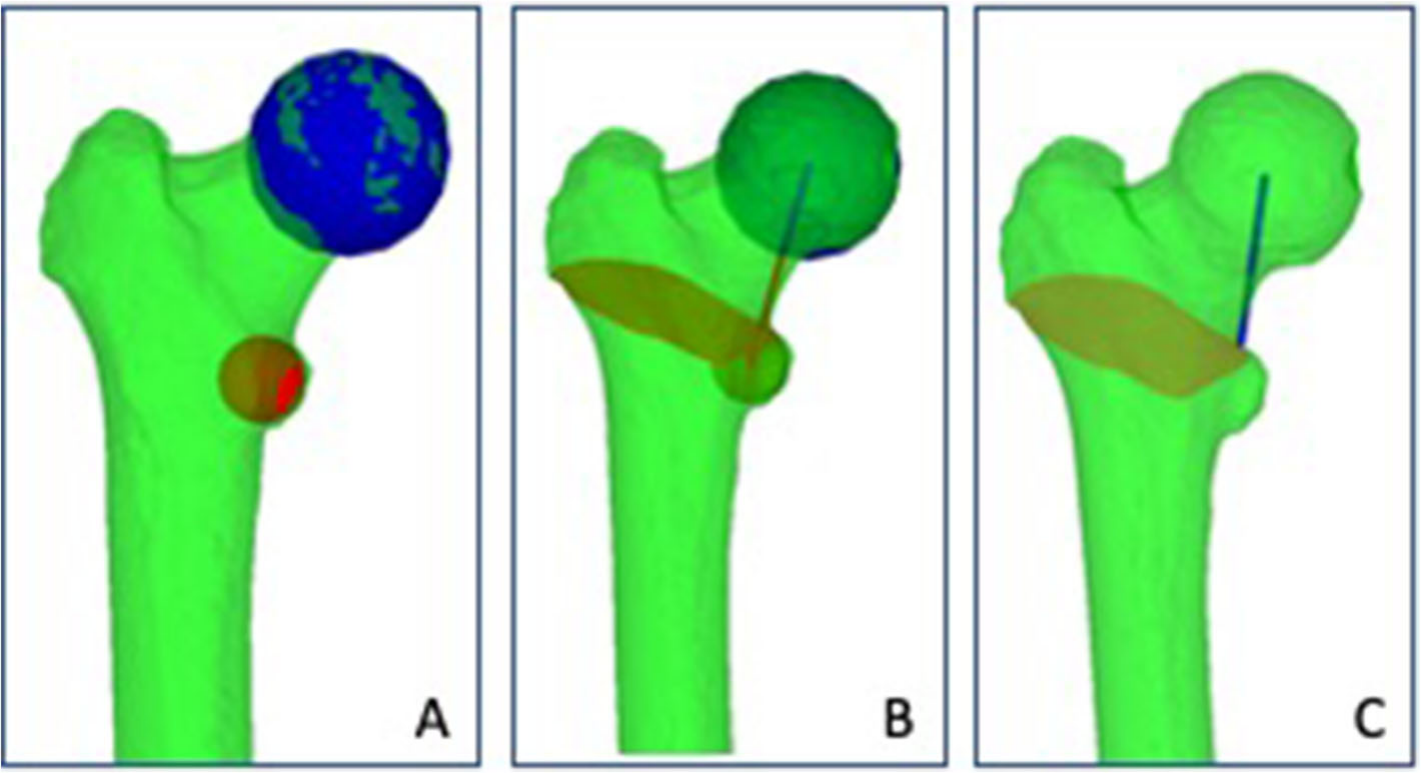
Measurement of the LTFHD. **a** Blue sphere: FHC; Red Sphere: LTC. **b** Red plane: Intersection of the 3D surface model of the femur and a plane with the LTFHA (defined as the axis connecting the LTC and the FHC) as plane normal. **c** UBLT was defined as the point on the border of this intersection with the smallest distance to the line connecting the FHC and the LTC. Blue line connecting the UBLT and the FHC equals the LTFHD

The LTFHD was measured as follows:

1. The femoral head center (FHC) was defined as the center of a best-fit sphere calculated form all surface points of the femoral head. The femoral head diameter was defined as the diameter of the sphere (FHD). (Fig. 2a)

2. Thereafter, a sphere with a diameter of 20 mm was placed in the center of the lesser trochanter (LTC). (Fig. 2a)
3. An axis connecting the LTC and the FHC was defined (LTFHA). A plane with the LTFHA as plane normal was placed on the upper end of the sphere fitted in the LTC. The intersection between this plane and the 3-D surface model of the femur was generated. (Fig. 2b)
4. The point at the upper border of the lesser trochanter (UBLT) was then defined as the point at the border of the previously generated intersection with the smallest distance to the center of the line connecting the LTC and the FHC. (Fig. 2c)
5. The distance between the UBLT and the FHC was defined as the LTFHD. (Fig. 2c)

Due to manual digitization involvement for the determination of the PPC, FNA, FHC, the FHD and the LTC, all measurements were performed by two independent blinded observers (J.H. and A.H.) for evaluation of inter-observer reliabilities of the measurements.

### Statistical analysis

Continuous variables are reported as mean and standard deviation (SD). Inter-reader reliabilities of the measurements were evaluated with the interclass correlation coefficients (ICC) with a 2-way random-effects model for absolute agreement. Normality of distribution was tested using the Shapiro-Wilk test. Accordingly, the two-tailed paired t-test or Wilcoxon signed-rank test was applied to assess side-to-side differences based on the delta of the left minus right femur. Further, to assess a potential correlation between the FHD and the LTFHD, the ratio between the LTFHD and the FHD of the corresponding sides were calculated. Univariate linear regression models were established, to measure a possible two-way linear association between side-to-side differences of the geometry of the proximal femur. Statistical analysis was performed using SPSS Statistics (SPSS, IBM Corporation, 1 New Orchard Road Armonk, New York 10,504 − 1722, USA). *P*-values > 0.05 were considered statistically significant.

## Results

Interobserver ICC ranged from 0.89 to 0.98. The interobserver ICC was 1 for the femoral leg length (Table 1). A statistically significant side-to-side-difference was found for the femoral head diameter (*p* = 0.03) and the LTFHD (*p* = 0.02) The mean absolute side-difference for LTFHD was 1.6 ± 1.4mm with a correlation coefficient (r) between sides of 0.93 (*p* < 0.01). Further information regarding side-to-side differences of the femoral anatomy are listed in Table 1. In total, 70 % of the paired femora showed an absolute side-difference in LTFHD < 2mm, 22 % between 2 and 4mm, and 8 % > 4mm (Table 2). Side-difference in LTFHD was not correlated to side-difference in femoral antetorsion (*r* = 0.07, *p* = 0.64) and femoral length (*r* = 0.4, *p* = 0.74). Regarding the ratio of the FHD and the LTFHD, a mean ratio of 1.16 ± 0.08 with a correlation coefficient (r) of 0.72 (*p* < 0.01) was found between these parameters. A subgroup analysis comparing femora with an absolute side-difference of more than 4mm revealed no significant differences in patient demographics.

**Table 1:** Summary of Side-to-Side Differences in paired femora. SD, Standard deviation; FHD, femoral head diameter; LTFHD, lesser trochanter femoral head distance. * indicates statistically significance difference (*p* < 0.05)

	Total (*n* = 100) Mean (SD)	Left (*n* = 50) Mean (SD)	Right (*n* = 50) Mean (SD)	Inter-observer interclass correlation	Absolute Difference Mean (range)	*P*-value of Side-Difference
**Femoral length (mm)**	**466.0 (29.3)**	**466.3 (29.9)**	**465.7 (28.9)**	**1**	**3.4 (01–14.9)**	**0.36**
**Femoral Antetorsion (°)**	**10.0 (7.6)**	**10.7 (7.4)**	**9.4 (7.8)**	**0.93**	**4.0 (0.1–13.0)**	**0.06**
**FHD (mm)**	**47.6 (3.9)**	**47.5 (3.9)**	**47.8 (4.0)**	**0.89**	**0.4 (0–3)**	0.03*
**LTFHD (mm)**	**55.0 (5.3)**	**55.4 (5.4)**	**54.7 (5.2)**	**0.98**	**1.6 (0.1–5.5)**	0.02*

**Table 2:** Range of absolute difference in LTFHD and femoral length

	Absolute Side-Difference in LTFHD	**Absolute Side-Difference in femoral length**	
**< 2mm**	**70 %**	**30 %**	
**2–4 mm**	**22 %**	**38 %**	
**> 4mm**	**8 %**	**32 %**	

## Discussion

The key finding of the current study was a statistically significant side-difference of the LTFHD (*p* = 0.02) with a mean absolute difference of 1.6 +/- 1.4mm and a correlation coefficient (r) between sides of 0.93 (*p* < 0.01). However, 8 % of the specimens showed an absolute side-difference of more than 4mm.

Side-to-side asymmetry of the femoral anatomy has been studied by various authors. Unnanuntana et al. used digital photographs of 200 cadaveric femora for a 2D analysis of different reference parameters frequently used to restore normal anatomy of the proximal femur during total hip replacement. In their study, they found an absolute side-difference of the LTFHD of 1.9 ± 1.8mm with a correlation coefficient between sides of 0.87 [14], which is in accordance with the results of the current study.

Other authors have attempted to develop alternative methods to predict the LTFHD than using measurements of the contralateral side. Polishchuk et al. retrospectively reviewed demographic and radiographic variables of 258 patients undergoing unilateral total hip arthroplasty or hemiarthroplasty. In their study, they found various variables including relative neck length, age, height, weight, gender and race to significantly correlate with LTFHD. Based on their findings, they derived an equation to predict LTFHD. However, although the average predicted LTFHD was within 2.9 mm of the intra-operatively measured values, the difference between the measured and predicted value varied up to 1.5 cm, and the prediction only had a fair ICC of 0.65 [25]. Sproul et al. investigated a potential correlation of the FHD and the LTFHD. After examining the anatomy of 34 cadaveric femora, they concluded, that the LTFHD can be predicted by the following equation: FHD (mm) x 1.035 = LTFHD. However, their study entailed only a relatively small number of specimens and the correlation between the FHD and the LTFHD was only moderate (*r*^2^ = 0.46) [15]. Consistent with their findings, Unnanuntana et al. reported an average ratio between the LTFHD and the FHD of 1.01 ± 0.12 measured on plane photographs [14]. In our study, we found a slightly higher average ratio between the LTFHD and the FHD of 1.16 ± 0.08 with a correlation coefficient (r) between these parameters of 0.72, which might be explained by the 3D measurement method of the FHD as well as the LTFHD. Taken together, these data suggest the FHD as an alternative option to predict the LTFHD, if measurement of the contralateral LTFHD is not possible due to distorted anatomy on both hips.

Atkinson et al. analyzed CT scans of 100 consecutive patients undergoing hip resurfacing arthroplasty surgery for hip osteoarthritis to find sex differences in hip morphology. In their study, the found no side-to-side difference for femoral antetorsion, femoral neck angle and femoral offset [26]. Using CT-based 3D femoral models, Dimitriou et al. compared the anatomy of 122 paired femora. Contrary to the findings of Atkinson et al., they demonstrated a statistically significant average side-difference in femoral antetorsion of 4.3° (range 0° − 17.3°). Similarly, the current study shows a mean side-difference of 4.0° (range 0.1° − 13.0°) for femoral antetorsion, although our findings were not statistically significant (*p* = 0.1). Together, these data suggest a high potential asymmetry of the femoral antetorsion, which should be considered whenever the contralateral femur is used as a reference for correction or restoration of an advantageous anatomy. Additionally, they showed a mean absolute side-difference of 2.5 ± 2.1mm for the horizontal offset, defined as the perpendicular distance between the anatomical femoral axis and the FHD, and 2.1mm ± 1.8mm for vertical offset, which was defined as the vertical distance between a line connecting the tip of the lesser trochanter and the FHC. Although they did not measure the LTFHD, these findings indicate a potential side-difference of this parameter.

Although the current study demonstrated a statistically significant absolute side-difference of the average LTFHD of 1.6 ± 1.4 mm, this might not be of clinical relevance, since the resulting templating error for THA would be small and the majority of patients with minor LLD after THA have no or only few symptoms [2] However, osseous asymmetry might also occur in the pelvis. Therefore, during templating of total hip reconstruction, simple mirroring of the contralateral LTFHD without considering potential differences in pelvic morphology might lead to unsatisfiable results, since unrecognized concomitant femoral and pelvic asymmetry might cumulate and lead to symptomatic global leg length discrepancy. Furthermore, although side-differences in the LTFHD were not correlated with side-differences in femoral antetorsion, the intraoperatively measured LTFHD most likely depends on the torsion of the inserted femoral stem during THA. Consequently, alteration of the natural femoral antetorsion during THA might lead to variation of the intraarticular leg length compared to the preoperative templating. However, further studies are needed to quantify the impact of the torsion of the femoral stem on the intraoperatively measured LTFHD.

This study should be interpreted in light of its potential limitations. First, only limited demographic data were available of the examined specimens. Therefore, the impact of potential confounders such as ethnics and race could not be investigated. Second, only femora of subjects without any macroscopic pathology of the hip and the femur were included. Yet, the majority of patients requiring surgery suffer from altered anatomy due to degenerative or traumatic changes, which must be considered when THA is planned. Third, only femoral asymmetry was investigated in the current study. However, to achieve leg length equality, global leg length discrepancy and asymmetry of the pelvis must be taken into account, which was not assessed with the current study. Nonetheless, the current study provides the first 3D analysis of side-differences of the LTFHD. Therefore, our findings are free of projection errors resulting from measurements on plane radiographs, thus reflecting the intraoperative conditions more accurately. Furthermore, due to the standardized measurement method, the presented data demonstrated an excellent interclass ICC of 0.98 for measurement of the LTFHD. Lastly, no plane radiographs of the investigated specimens were available for analysis, thus a comparison of the 3D CT-based measurement of the LTFHD with measurements from 2D plane radiographs was not possible. Since measurements from plane radiographs are strongly influenced by the position of the patient (e.g. rotation/flexion of the hip), the findings from the current study might not necessarily correlate with measurements on plane radiograph. However, LTFHD is not only used for planning of total hip arthroplasty, but also for intraoperative validation of the transfer of the preoperative planning. The measurements of the LTFHD performed during surgery reflect the 3D CT-based measurements performed in this study. Thus, side-to-side difference, which remain unnoticed during preoperative planning on plane radiographs might lead to unintended Leg-Length-discrepancy after total hip arthroplasty.

## Conclusions

The LTFHD is a reliable reference parameter for preoperative templating and intraoperative validation during THA with only minimal side-to-side difference and a high correlation between sides (r = 0.93, p = < 0.01). However, 8 % of the current cohort showed a difference in LTFHD of more than 4mm, which should be anticipated during THA to avoid unsatisfiable results.

## Data Availability

The datasets generated and analysed during the current study are available from the corresponding author on reasonable request.
